# An elimination method for isolated meshes in a road network considering stroke edge feature

**DOI:** 10.1371/journal.pone.0239828

**Published:** 2020-11-30

**Authors:** Chengming Li, Wei Wu, Pengda Wu, Jie Yin, Peipei Guo

**Affiliations:** 1 College of Geomatics, Shandong University of Science and Technology, Qingdao, China; 2 National Engineering Laboratory for Integrated Aero-Space-Ground-Ocean Big Data Application Technology, Xi’an, China; 3 Chinese Academy of Surveying and Mapping, Beijing, China; China University of Geosciences, CHINA

## Abstract

The road network is the skeletal element of topographic maps at different scales. In general, urban roads are connected by road segments, thus forming a series of road meshes. Mesh elimination is a key step in evaluating the importance of roads during the road network data management and a prerequisite to the implementation of continuous multiscale spatial representation of road networks. The existing mesh-based method is an advanced road elimination method whereby meshes with the largest density are sequentially selected and road segments with the least importance in each mesh are eliminated. However, the road connectivity and integrity may be destroyed in specific areas by this method because some eliminated road segments could be located in the middle of road strokes. Therefore, this paper proposed an elimination method for isolated meshes in a road network considering stroke edge feature. First, small meshes were identified by using mesh density thresholds, which can be obtained by the sample data statistical algorithm. Thereafter, the small meshes related to the edge segments of road strokes were taken out and defined as stroke edge meshes, and the remaining small meshes were defined as stroke non-edge meshes. Second, by computing the mesh density of all stroke edge meshes, the mesh with the largest density was selected as the starting mesh, and the least important edge segment in the mesh was eliminated. The difference between the existing mesh-based method and the proposed method is that the starting mesh is a stroke edge mesh, not any given small mesh, and the eliminated segment is just only one of edge segments of strokes not chosen from among all segments. Third, mesh elimination was implemented by iteratively processing the stroke edge meshes with the largest mesh density until all of them were eliminated and their mesh density exceeded the threshold. The stroke non-edge meshes were directly preserved. Finally, a 1:10,000 topographic road map of an area in Jiangsu Province of China was used for validation. The experimental results show that for all stroke non-edge meshes and 23% of the stroke edge meshes, compared to the mesh-based method, the road stroke connectivity and integrity of road strokes were better preserved by the proposed method, and the remaining 77% of the elimination results for the stroke edge meshes were the same under the two methods.

## Introduction

Road network is the skeletal element of topographic maps at different scales. In many geographic information systems applications related to earth sciences, transportation and urban planning, multi-representation road network databases obtained through cartography generalization are needed for conducting analyses, evaluations and presentations with varying levels of detail [1, 2]. In a database, a road network consists of a number of road intersections and road segments, and the road segments usually formed a series of road meshes. In considering the constructing of multi-representation databases from original road network, the primary process is mesh elimination [3–5], which is a process to eliminate less important roads while the essential topological, geometric and spatial structure characteristics of a road network are preserved when the map scale get smaller [6–8].

The objective of mesh elimination is not only to simplify and reduce the complexity of the road network, but also to detect and preserve the original road network characteristics [7], especially for the maintaining of topological and spatial structure characteristics, which are indispensable in the field of traffic flow forecast, course planning and travel navigation [9, 10]. To get a satisfactory elimination result, an enormous amount of researches have been carried out worldwide. Existing methods can be divided into three categories. The first one is based on graph theory, by which the topologic structure of a road network is captured by a primary graph [11, 12]. In a related study to primary graph theory, Thomson et al. [13, 14] recommended a stroke-based method by considering the good continuation principle in Gestalt theory. However, it is not easy for the above two approaches to characterize the structural properties of a road network [15]. To address this issue, Hu et al. [16] and Chen et al. [17] proposed a mesh-based method, in which the density characteristics of road networks are well described, and many scholars have pointed out that this method is a common, advanced and powerful model to conduct road mesh elimination work in dense road areas [18–20]. However, the connectivity and integrity of roads may be destroyed in some special areas by using this method because some eliminated road segments are located in the middle of roads. Hence, this paper proposed an elimination method for isolated meshes in a road network considering stroke edge feature. The objective and contribution of the proposed method is to obtain more reasonable mesh elimination results, in which not only the spatial shape and density characteristics of the road network were reflected accurately, but also the connectivity and integrity of the roads themselves were preserved.

The paper is organized as follows. Section 2 presents the common and advanced mesh-based road elimination methods and analyzes their limitations. Section 3 introduces the elimination method for isolated meshes considering stroke edge feature. Section 4 provides a series of experiments that were conducted to validate the reliability and superiority of the proposed method. The conclusions are described in section 5.

## Literature review

### Existing methods for road mesh elimination

Road mesh elimination in road networks has been the subject of extensive studies. Mackaness et al. [11] and Wanning et al. [12] proposed elimination methods based on the graph theory, which easily organize road network data and preserve the topological relationship of road networks during the elimination process. Nevertheless, these methods cannot describe the road shape and road network structure. By introducing the principle of good continuation in the Gestalt theory of visual perception, Thomson et al. [13, 14] proposed a mesh elimination method based on stroke features. In this method, roads were eliminated based on the importance of their strokes. To improve the accuracy of elimination, Liu et al. [21, 22] considered the length, connectivity and average arc density to calculate the stroke importance, and Zhou et al. [23] employed the connectivity, centrality, road class, type and other semantic information of strokes in the importance calculation. Stroke-based elimination methods can effectively simulate the visual continuity similar to human cartographers [24]. Nonetheless, these methods fail to consider the distribution density of roads, which is an important constraint [15].

To address this issue, Hu et al. [16] and Chen et al. [17] proposed road mesh elimination methods based on the mesh density of a road network. The so-called mesh is a closed region surrounded by several road segments. The density of each mesh in the road network is calculated by dividing mesh perimeter by mesh area. For a given target scale, the mesh density threshold is firstly determined by using the sample data statistical algorithm [17], then all small meshes, whose mesh densities are below the threshold value, are identified. For each small mesh, the road segment with the least importance is eliminated. The elimination starts with the small mesh with the largest mesh density and iteratively processes until there is no small mesh in the road network. This method can suitably describe and summarize the density characteristics of road networks, and many scholars have pointed out that this method is a common, advanced and powerful model to conduct road mesh elimination work in dense road areas [18–20], such as urban areas.

### Limitations of the existing methods

**Fig 1:**
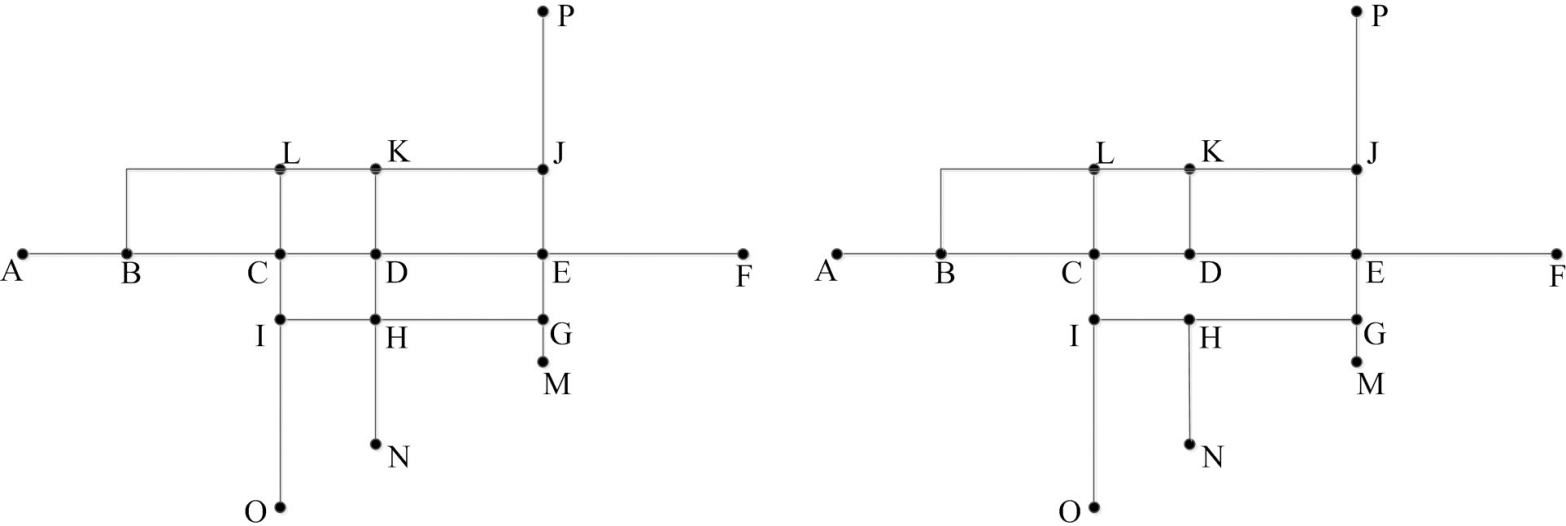
Schematic diagram of the limitations of the common and advanced mesh-based method. (a) original road network in which mesh CDHI is the small mesh with the largest density, and road segment DH is the segment with least importance and (b) segment DH was eliminated by the mesh-based method, leading to the loss of the connectivity and integrity of road KDHN and a new dangling segment HN is created.

The mesh-based method has been proven to reflect the overall spatial structure and local density variations of road networks [17]. Li and Zhou [19] applied this method to deal with areal pattern roads (i.e., road segments belonging to one or two meshes), while Wu [8] and Touya [18] also applied the mesh-based approach to road elimination in urban areas. However, if the road segment located in the middle of the road stroke is eliminated, the road stroke connectivity and integrity cannot be maintained, as shown in Fig 1.

## Materials and methods

**Fig 2:**
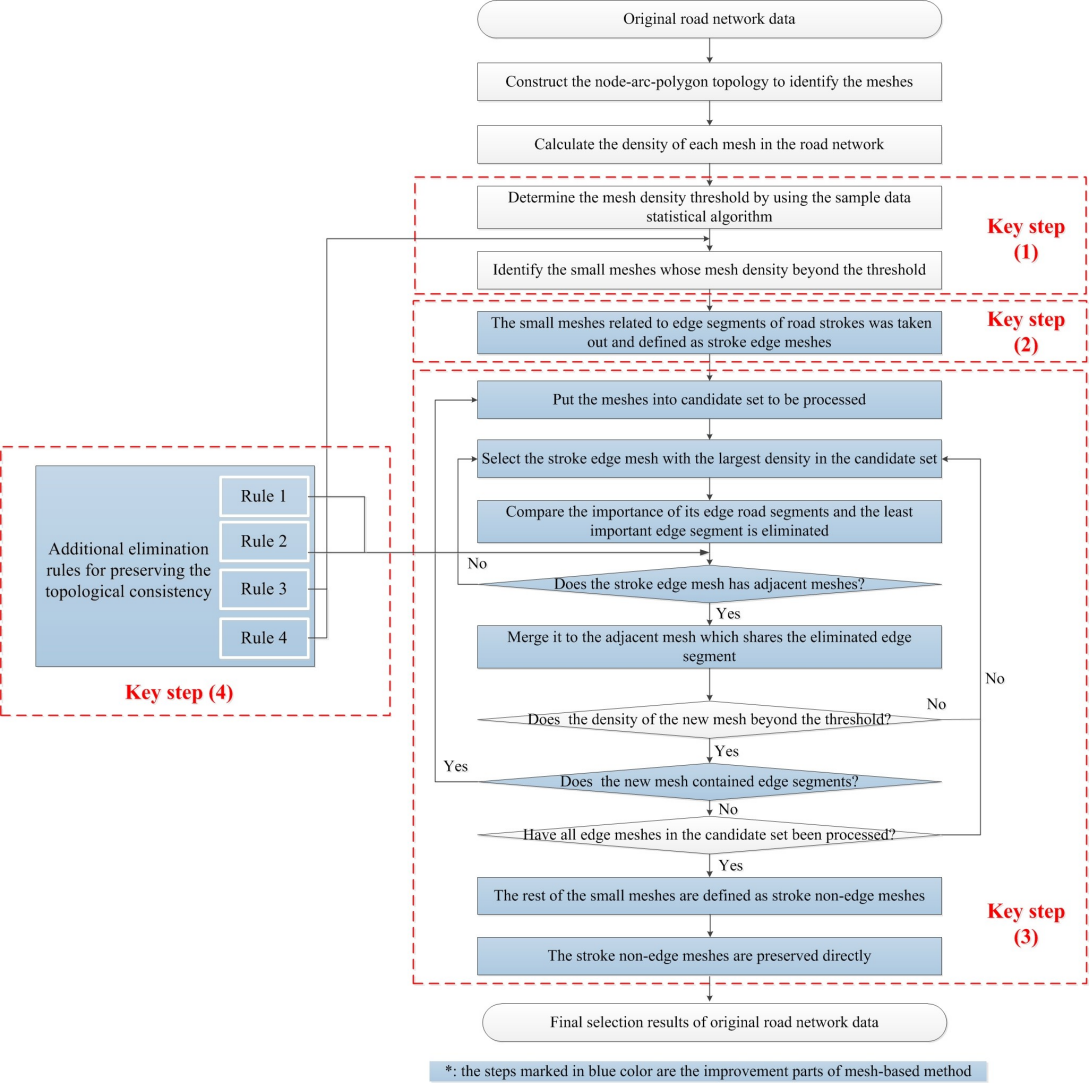
Detailed flowchart of the proposed method.

Aimed at the limitations of the existing advanced mesh-based method, an elimination method for isolated meshes in a road network considering stroke edge feature is proposed in this paper, which consists of the following four key steps: (1) Determination of the mesh density threshold value and classification of small meshes by using the sample data statistical algorithm; (2) Identification of the stroke edge features, including the edge segments of road strokes, stroke edge meshes and stroke non-edge meshes; (3) Progressive elimination algorithm, whereby mesh elimination is implemented by iteratively processing the stroke edge mesh with the largest mesh density until all stroke edge meshes with a mesh density greater than the mesh density threshold have been eliminated, and the stroke non-edge meshes are directly preserved; (4) Additional elimination rules for preserving the topological consistency of the road network. A detailed flowchart is shown in Fig 2.

### Determination of the mesh density threshold and classification of the small meshes

The mesh density threshold is a core parameter of the mesh-based approach. Hu et al. [16] and Chen et al. [17] proposed a threshold determination algorithm for the mesh density based on an empirical study, which is called the sample data statistical algorithm in this paper. The basic principle of this algorithm is as follows. First, the mesh density distribution curves for the sample data before and after road network processing are obtained and plotted, and each curve represents the relationship between the number of meshes and the mesh density. Second, comparing the two curves, the corresponding value of the split node at which the curves become notably different is regarded as the threshold. For different generalization scales, the threshold is also different, and the detailed calculation procedure was described in Hu et al. [16] and Chen et al. [17].

**Fig 3:**
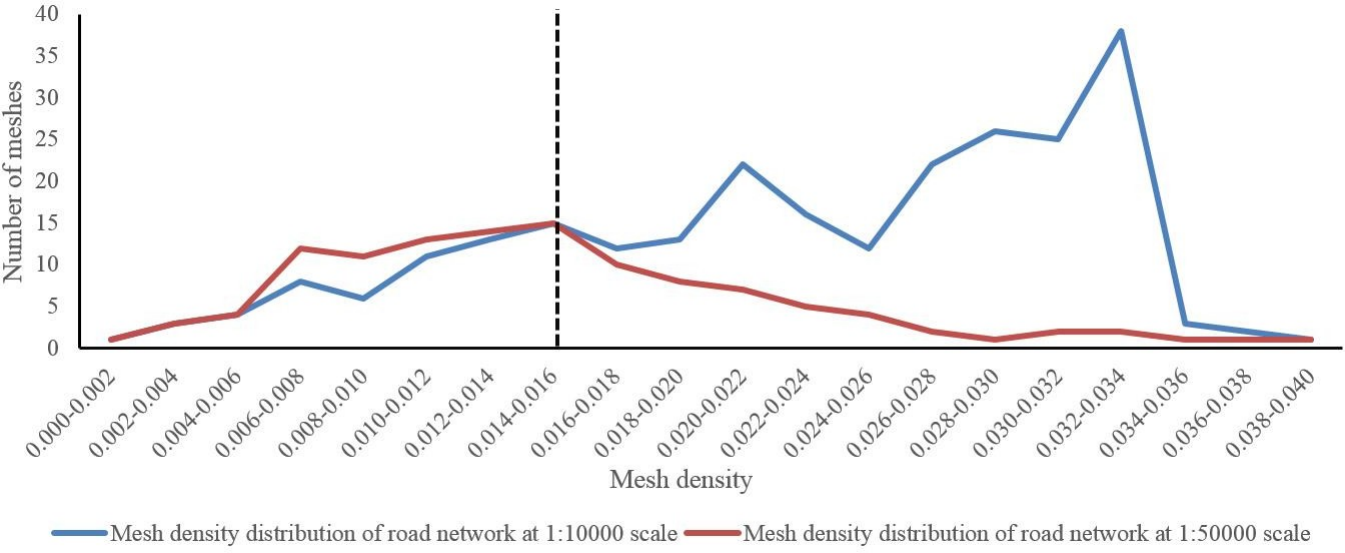
Determination of the most appropriate threshold for the mesh density.

In our paper, the sample data statistical algorithm is also adopted to calculate the mesh density threshold. To illustrate the calculation procedure, an example of a scale change from 1:10,000 to 1:50,000 was performed. The sample data of these two scales comes from the data in Section 4, and the mesh density distribution curves at the two scales is shown in Fig 3. When the mesh density equals 0.016 m/m^2^, the difference between the two curves begins to increase gradually. Therefore, 0.016 m/m^2^ is considered the mesh density threshold at the scale of 1:50,000 in this study. Correspondingly, meshes with a mesh density higher than the threshold are defined as small meshes.

### Identification of the stroke edge features

Observing the spatial characteristics of road networks at the different scales, it can be found that to preserve the spatial structure of the road network, all the small meshes will be eliminated in the mesh-based method. However, to maintain the road connectivity and integrity, iterative elimination of the meshes only involves the stroke edge meshes in our method, which first need to be identified and determined via their stroke edge features. Hence, stroke edge features are detected and adopted as a new constraint for mesh elimination in this paper.

The concept of the stroke is based on the principle of ‘Good Continuation’ in the theory of Gestalt psychology, which states that elements that appear to follow the same direction tend to be grouped together [13, 14]. In this study, strokes are built upon the arc-node topology structure of the road network considering the names and directions of the road segments at a junction. This process was described in detail in our previous study [25].

**Fig 4:**
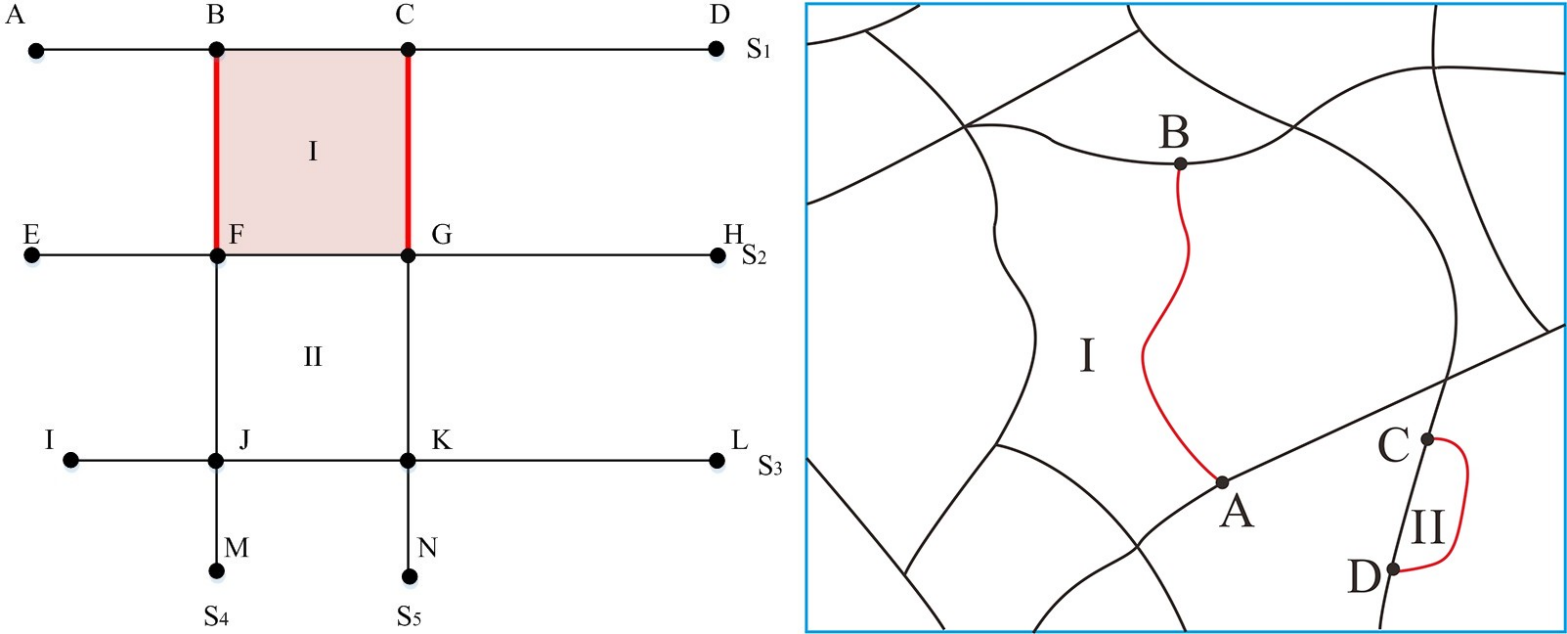
Schematic diagram of the edge segments and stroke edge meshes. (a) outermost area of the road network and (b) single-loop and connected areas inside the road network.

The following identification rules for a road network are defined to detect the stroke edge features:

1. Edge segment: for a road segment, if the number of intersection nodes between itself and other segments in the same road stroke is smaller than 2 (i.e., 0 or 1), this road segment is defined as an edge segment. As shown in Fig 4A, the road stroke S5 contains three road segments, i.e. CG, GK and KN. For the road segment CG, there is only one intersection node between it and the other two road segments, which is node G. Moreover, there are two intersection nodes between road segment GK and the other two road segments, which is nodes G and K, and there is one intersection node between road segment KN and the other two road segments, which is node K. Hence, the edge segments of road stroke S5 include CG and KN. Similarly, the edge segments of road stroke S1 include road segments AB and CD, the edge segments of road stroke S2 include EF and GH. It is worth noting that if there is a closed road whose start and end nodes coincide, it is also designated as an edge segment.
2. Stroke edge and non-edge meshes: the small meshes related to the edge segments are defined as stroke edge meshes, and correspondingly, the rest of the small meshes are defined as stroke non-edge meshes. For example, the mesh I in Fig 4A are stroke edge mesh because of the road segments BF and CG are edge segments, and the mesh II are stroke non-edge mesh because of there is no edge segment in this mesh. The fundamental difference between stroke edge mesh I and stroke non-edge mesh II is the occurrence of one or more edge segments. For the stroke edge mesh, an edge segment exists, and if it is deleted, the road connectivity will not be affected. In contrast, for the stroke non-edge meshes, edge segments do not exist, and should any of the road segments in the mesh be eliminated, the connectivity of the associated strokes will be surely affected.

In general, stroke edge meshes are primarily concentrated in three kinds of regions: the outermost area of the road network, the single-loop area inside the road network and the connected area formed by secondary roads. As depicted in Fig 4A, stroke edge mesh I is located in the outermost area of the road network, while in Fig 4B, road segment AB is an edge segment. Hence, mesh I, which contains this edge segment, is a stroke edge mesh located in a connected area formed by secondary roads. Road segment CD is also an edge segment. Hence, mesh II is a stroke edge mesh located in a single-loop area inside the road network.

### Progressive elimination algorithm

To implement the entire elimination process of the stroke edge meshes one at a time, not including the stroke non-edge meshes, a progressive elimination algorithm is proposed. The workflow for this algorithm is designed and is composed of the following novel steps:

Step 1: The stroke edge meshes are identified from the small meshes, and they are assigned to the candidate set to be processed.

Step 2: The stroke edge meshes in the candidate set are sorted in descending order of the mesh density, and the mesh with the largest density is first processed.

Step 3: The edge segments in the stroke edge mesh are marked, and the importance of each edge segment is calculated with Eq (1) [17]:

$$I=\max\left\{\omega_{1}{C,\omega }_{2}D_{S}{,\omega }_{3}L_{S}{,\omega }_{4}L\right\}$$ (1)

where I indicated the importance index of each edge segments, C is the normalized road class, Ds is the normalized degree of the stroke, Ls is the normalized length of the road stroke, and L is the normalized length of the road segment, max{*} is a function to determine the importance of the edge segments, *ω_i_*.(i = 1, 2, 3, 4) represents the weight of each parameter. Before the calculation, these four parameters are normalized to (0, 1) through min-max normalization algorithm, and *ω_1_, ω_2_, ω_3_, ω_4_* are set to 10^4^, 10^3^, 10^2^, 10, respectively.

Step 4: The least important edge segment in this stroke edge mesh is eliminated.

Step 5: It is determined whether this stroke edge mesh has adjacent meshes. If it does, it is merged with the adjacent mesh that shares the eliminated edge segment with this stroke edge mesh. Otherwise, the algorithm proceeds with step 2.

Step 6: The density of the new mesh is calculated, and it is compared to the threshold. If it exceeds the threshold and contains edge segments, it is added to the candidate set, but if it does not exceed the threshold, the algorithm proceeds with the next step.

Step 7: Identify the small meshes adjacent to aforementioned processed stroke edge mesh, and determine it is or not the newly forms stroke edge mesh, if it is, update it into the candidate set.

Step 8: Steps 2–7 are repeated, and the algorithm ends when all the stroke edge meshes in the candidate set have been processed.

**Fig 5:**
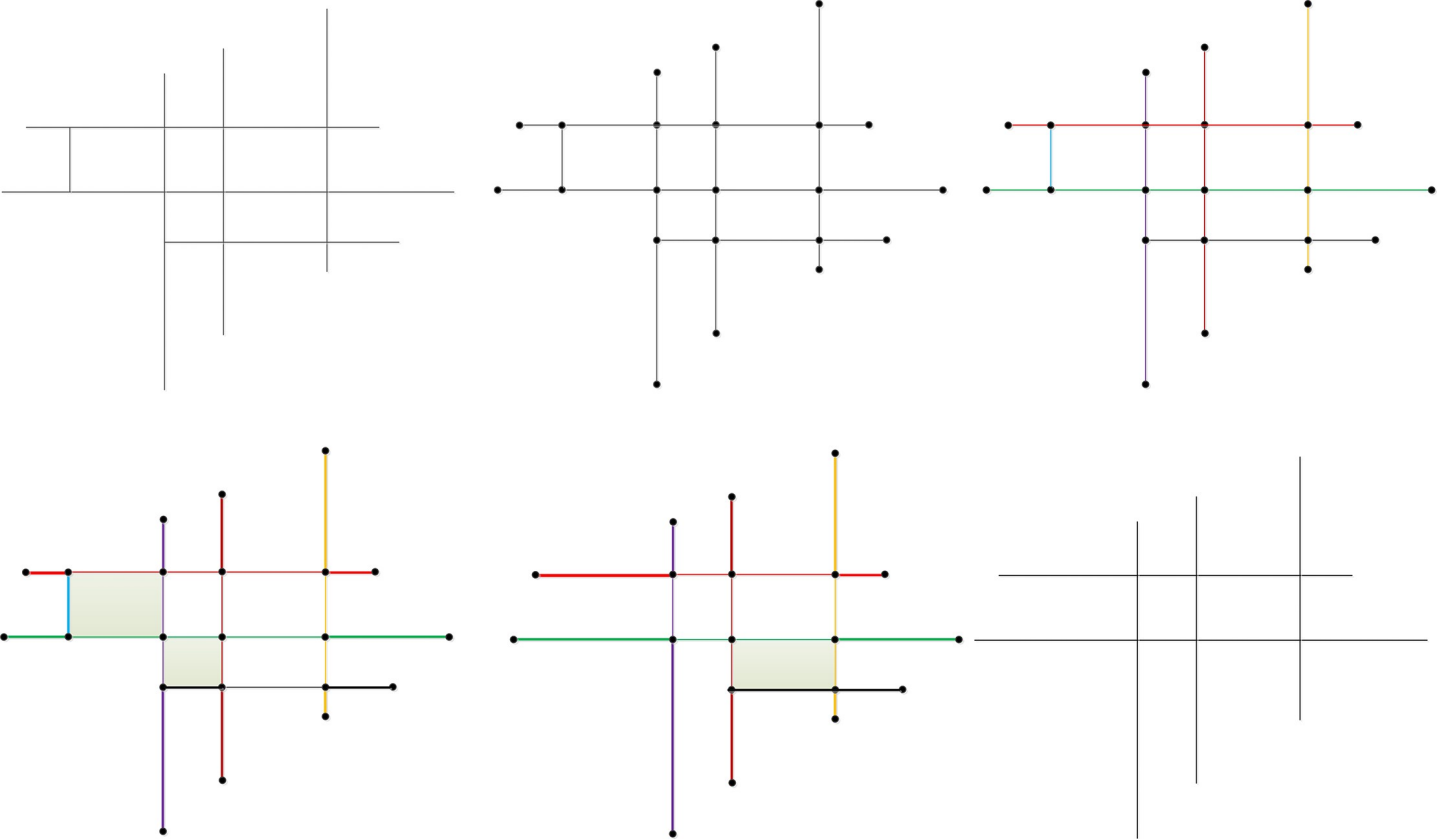
Schematic diagrams of the proposed algorithm. (a) original road network, assuming the meshes in the original data are all small meshes, (b) constructing the node-arc-polygon topology, and the semantic information of the road is recorded by the topology structure, (c) constructing the road strokes by considering the name and directions consistency of the road segments, (d) identifying the edge segments (the road segments which are highlighted) and stroke edge meshes (the filled meshes); (e) eliminating the edge segments in the stroke edge meshes and identifying the newly formed stroke edge mesh (the filled mesh) and (f) final elimination result of original road network.

The schematic diagrams of the proposed algorithm is shown in Fig 5 to clearly explain the overall process.

### Additional preservation of the topological consistency

The order of parameters reflecting the importance of the road segments in step 3 represents the priority in road mesh elimination. In addition, to coordinate the topological relationship between roads, a number of rules should be followed in the elimination process. Some of the rules adopted in this study are formulated as follows:

Rule 1: A dangling road segment will simultaneously be eliminated if its connected edge segment is eliminated.

Rule 2: If a new dangling road segment is created after an edge segment is eliminated, the dangling road segment will then also be eliminated.

Rule 3: A road segment connecting important features such as a dock will be preserved.

Rule 4: Before the identification of stroke edge features, dangling road segments with lengths smaller than the threshold will be marked and not considered during the identification procedure. The threshold value can be obtained from reference [19].

For the above four rules, Rule 1 and Rule 2 are applied in step 4 of the progressive elimination algorithm, Rule 3 is applied before step 1, the name of each road segment is recorded in the topological structure, Rule 4 is applied before step 1.

## Results and discussion

### Experimental data and environment

**Fig 6:**
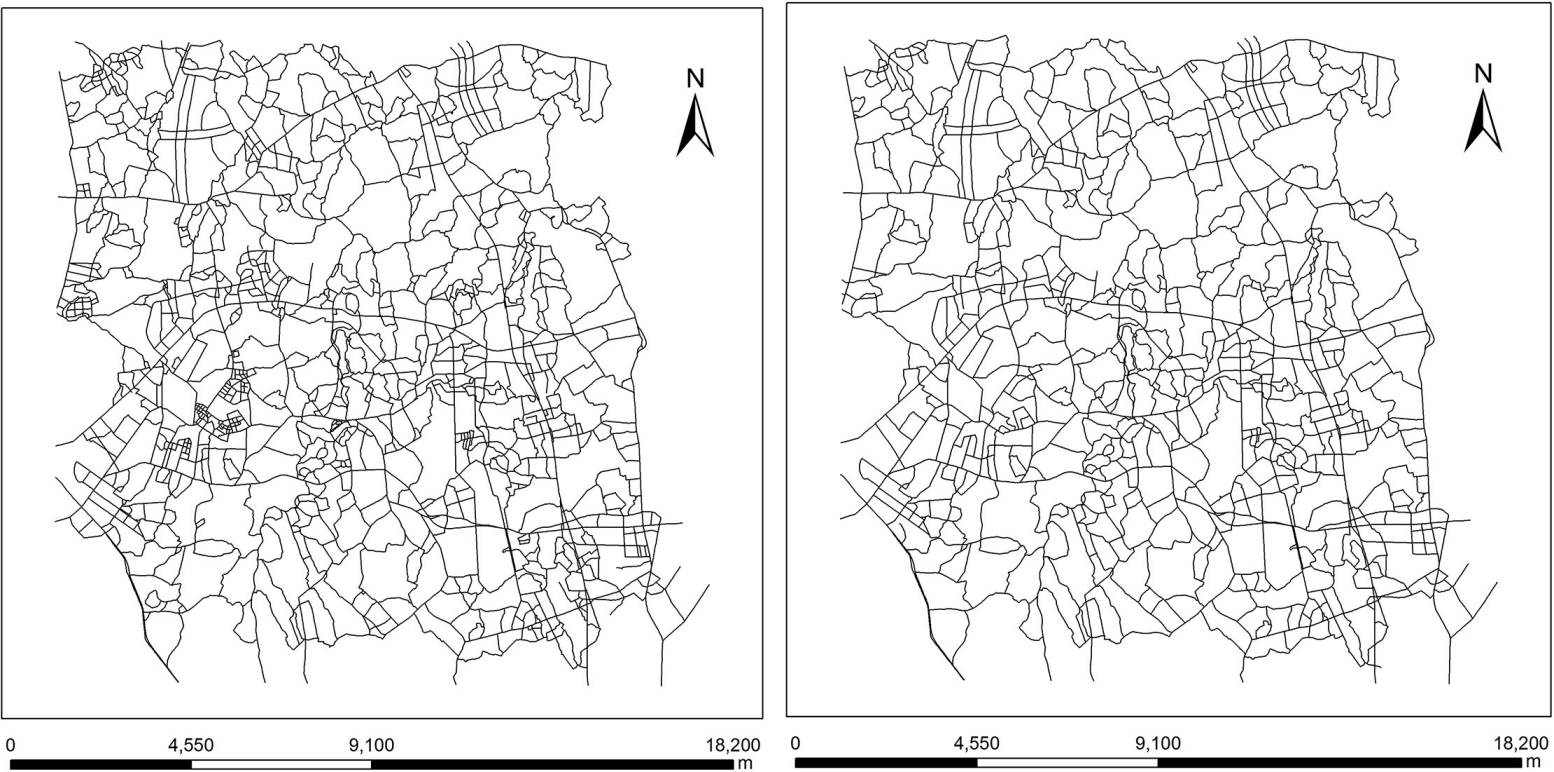
Experimental data. (a) 1:10,000 original road data, (b) 1:50,000 sample data.

The elimination method for isolated meshes in a road network considering stroke edge feature (hereafter called stroke edge-based method) in this paper was embedded into the WJ-III mapping workstation developed by the Chinese Academy of Surveying and Mapping (CASM). The reliability and superiority of the new stroke edge-based method was experimentally validated via a comparison to the common and advanced mesh-based method proposed by Chen et al. [17]. The experimental data are obtained from a 1:10,000 topographic road map of an area in Jiangsu Province in China. The copyright of the experimental data and result data belongs to the authors, and the data are available at: https://github.com/wpd2017/ RoadNetworkMeshElimination.git. The spatial scope of the experimental data is 14.91×15.67 km^2^, with a total of 1064 road objects, and the elimination target scale is 1:50,000. The number of road segments should be eliminated from scales 1: 10,000 to 1: 50,000 is determined by the mesh density threshold, which is calculated by the sample data statistical algorithm. The sample data at the two scales are shown in Fig 6, and the final threshold was set to 0.016 m/m^2^, which means that meshes with a mesh density higher than 0.016 m/m^2^ are defined as small meshes, and they are meshes that need to be processed.

The operating environment of the software system is a Windows 7 64-bit operating system with an Intel Core I7-3770 CPU, at a main frequency of 3.2 GHz, with 16 GB of memory and a 1024-GB solid-state hard disk.

The time required by the mesh-based and stroke edge-based methods in this paper to process the experimental data was recorded, which is 0.904 and 0.964 s, respectively.

### Comparative analysis of the eliminated meshes

**Fig 7:**
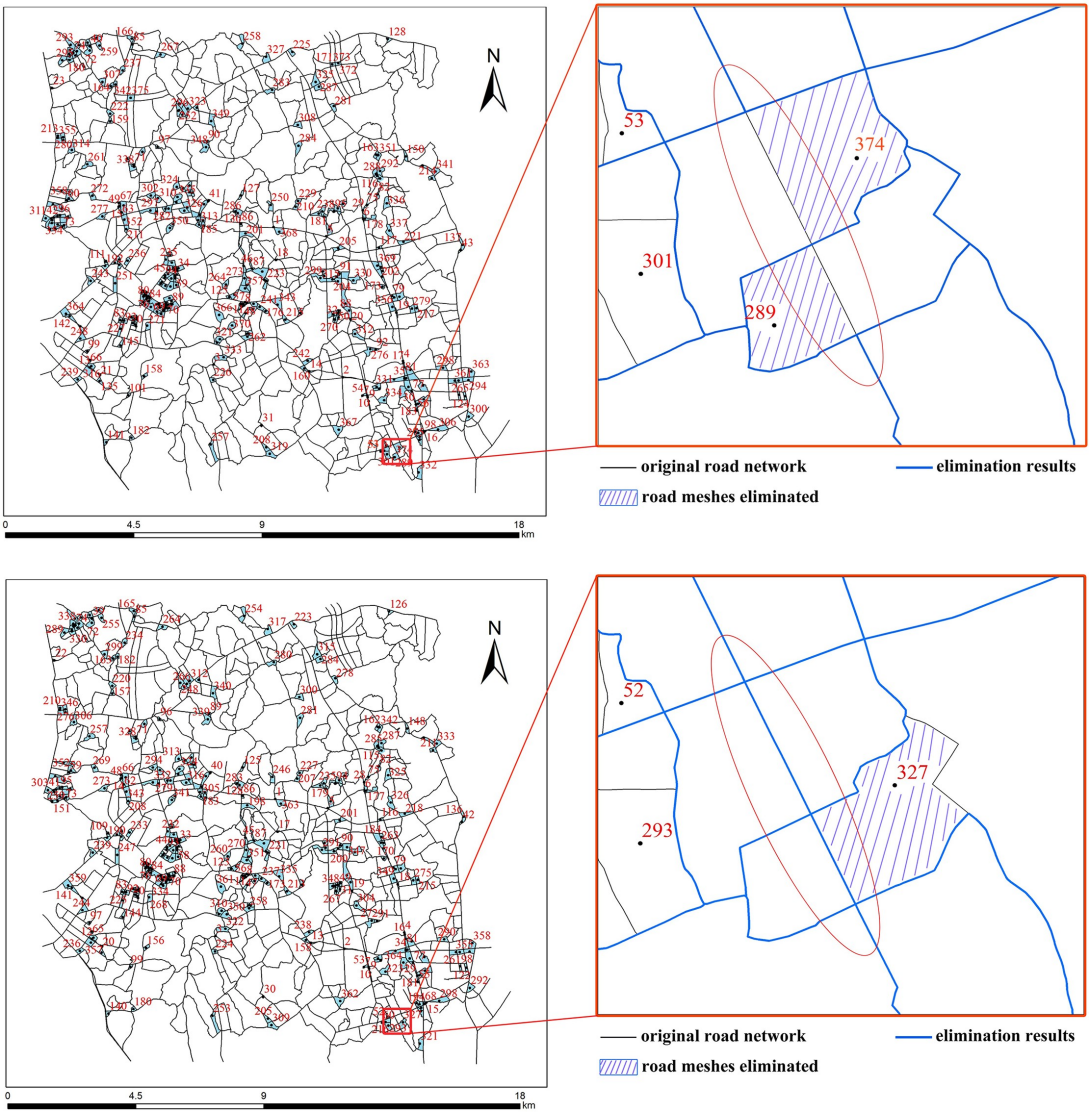
Comparative analysis of the mesh elimination order. (a) elimination order of the mesh-based method and (b) elimination order of the stroke edge-based method.

Fig 7A and 7B illustrate the mesh elimination order (marked in red) of the experimental road network data obtained using the mesh-based and stroke edge-based methods, respectively. As depicted in Fig 7, the elimination orders calculated by the two methods are completely different. A total of 376 small meshes were identified and eliminated in descending order of the mesh density by the mesh-based method. In contrast, 365 stroke edge meshes of the 376 small meshes were eliminated by the stroke edge-based method, and 11 stroke non-edge meshes were preserved. Local road region is further selected to exaggerate to show detailed information, and it can be found that the mesh elimination order directly affects the elimination results. As depicted in Fig 7A, mesh Nos. 289 and 304 were selected as the elimination object by the mesh-based method, and they were merged with their adjacent mesh respectively, which leading to the connectivity of the road in red ellipse was destroyed. In contrast, as depicted in Fig 7B, mesh No. 327 was selected as the elimination object by the stroke edge-based method, mesh Nos. 289 and 304 were not processed because they belong to stroke non-edge meshes. The edge segment in mesh No. 327 was eliminated, and the connectivity of the road in red ellipse was preserved. This finding suggests that the stroke edge-based method can better maintain the connectivity and integrity of the roads by adjusting the mesh elimination order.

### Quantitative analysis of the mesh elimination results

According to the three basic statistical indexes, such as the number of dangling roads and meshes and the total area of the meshes, the mesh elimination results from 1:10,000 to 1:50,000 by using the two different methods, as well as the 1:50,000 standard map and 1:10,000 original map, were obtained and compared, as listed in Table 1.

**Table 1:** Comparative analysis of the three basic statistical indexes.

Data	Dangling roads	Number of meshes	Total area of the meshes (km^2^)
**1:10,000 original map**	43	903	192.99
**1:50,000 standard map**	40	555	192.78
**Elimination result of the mesh-based method**	48	542	192.65
**Elimination result of the stroke edge-based method**	41	553	192.65

As indicated in Table 1, the number of dangling roads in the 1:10,000 original map and the 1:50,000 standard map is 43 and 40, respectively, revealing that 3 dangling roads were eliminated by a human cartographer during the mesh elimination process. First, compared to the mesh elimination result of the mesh-based method, which preserves 48 dangling roads, the stroke edge-based method only retains 41, which is closer to the number of eliminated roads by the human cartographer. Second, the number of preserved meshes of the stroke edge-based method is 11 larger than that of the mesh-based method. Similarly, the total number of meshes is closer to the elimination results of the human cartographer, thereby indirectly demonstrating that the distribution of road meshes is more reasonable. Finally, because of some road segments were eliminated, the original meshes they belonged to were disappeared, and if the remaining road segments in these mesh do not form new mesh, the total area of the meshes will be decreased. As shown in Table 1, the total areas of the meshes obtained by the two methods are both 192.65 km^2^, which indicates that although a large number of small meshes have been processed, the overall shape of the region formed by all preserved road meshes basically remains the same.

To further verify the reliability and superiority of the mesh elimination results, the maximum similarity and average connectivity are calculated and compared.

The calculation equation of the maximum similarity is as follows [20]:

$$\mathrm{Similarity}=\frac{A\cap B}{A+B‐A\cap B}$$ (2)

where A is the total length of the road network obtained by the mesh elimination method, B is the total length of the roads in the standard map at the same scale, and A∩B is the total length of the roads in common.

The average connectivity was proposed by Li and Zhou [19] to evaluate the overall connectivity characteristics of a road network, and the calculation equation is as follows:

$$AC=\frac{\sum_{i\in N}\;\sum_{j\in N,i\neq j}\partial_{\mathrm{ij}}}{N(N-1)}$$ (3)

where *N* is the number of network nodes and ∂_*ij*_ is 1 when there is a path from node *i* to node *j*. Otherwise, ∂_*ij*_ is 0. The value range of *AC* is [0, 1].

As seen from the above equation, the local road connectivity characteristic cannot be detected by *AC*. Therefore, we further improved this equation and defined a modified average connectivity in which the evaluation unit was changed to the road stroke. The modified calculation equation is as follows:

$$\mathrm{RAC}=\frac{\sum_{k=0}^{M}{AC}_{k}}{M}$$ (4)

where *RAC* is the sum of the average connectivity of each road stroke, *M* is the number of road strokes, and *AC_k_* is the average connectivity of the *k*^th^ road stroke.

The maximum similarity and modified average connectivity under each method are calculated according to Eqs (2) and (4), respectively, and the results are listed in Table 2.

**Table 2:** Comparison of the maximum similarity and the modified average connectivity.

Source scale	Target scale	Mesh elimination method	Maximum similarity (%)	Modified average connectivity
1:10000	1:50000	Mesh-based method	89.52	0.95
		Stroke edge-based method	91.64	1.00

As indicated in Table 2, compared to the mesh-based method, the maximum similarity between the results of the stroke edge-based method and the 1:50,000 standard map is up to 91.64%, which is slightly higher than the value of 89.52% for the mesh-based method. Additionally, the modified average connectivity of the mesh elimination results obtained by our method is 1, which indicates that this method effectively preserves the road connectivity for all road strokes, does not split any road stroke and does not produce any isolated segments. Conversely, the modified average connectivity of the mesh elimination results obtained by the mesh-based method is 0.95, indicating that some road strokes are split, and the road connectivity of these road strokes was destroyed.

### Visual analyses of typical regions

**Fig 8:**
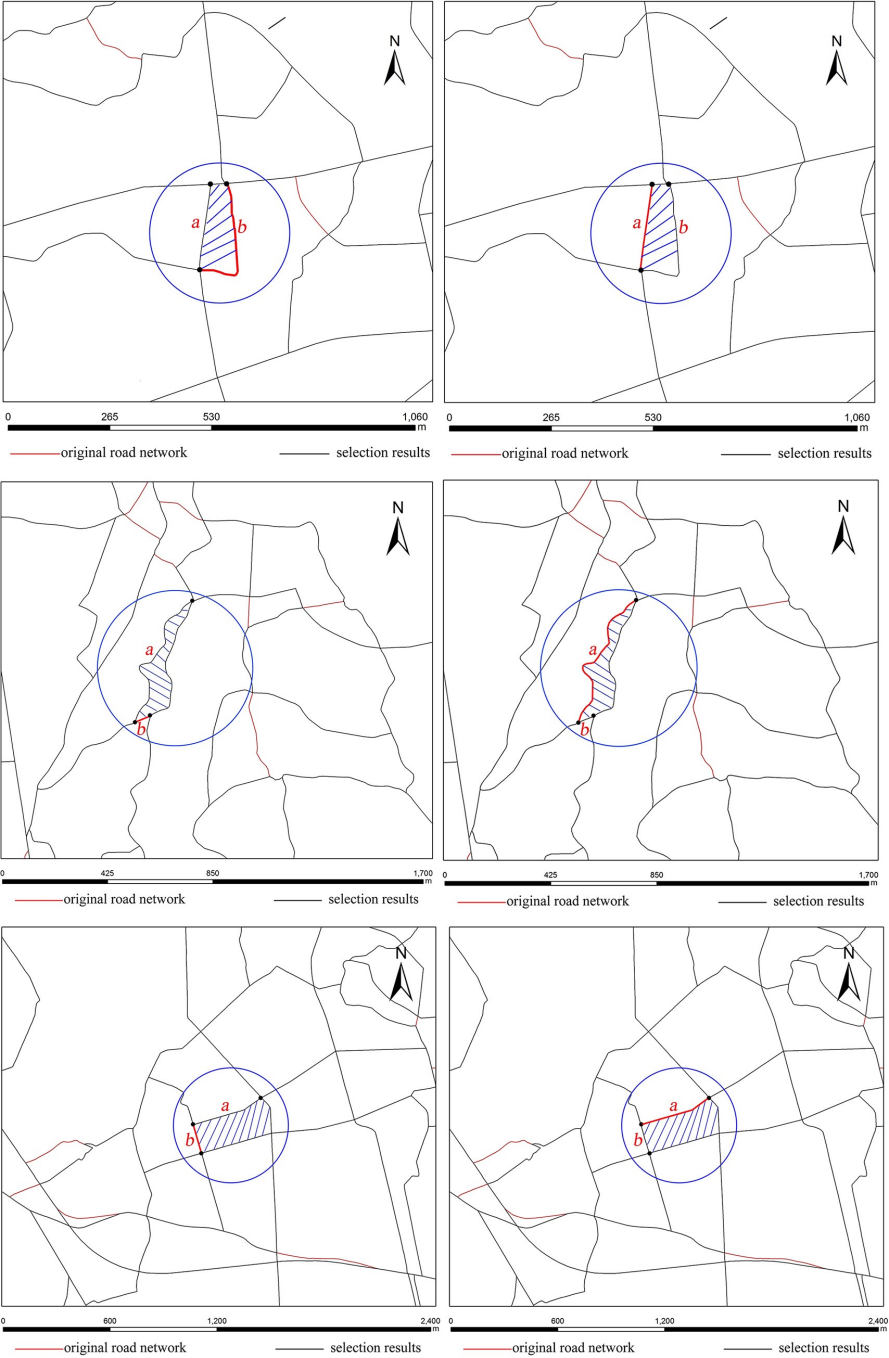
Comparison of the three typical areas for the stroke edge meshes with different elimination results. (a) Elimination result for the smallest mesh by the mesh-based method, (b) elimination result for the smallest mesh by the stroke edge-based method, (c) elimination result for the modest mesh by the mesh-based method, (d) elimination result for the modest mesh by the stroke edge-based method, (e) elimination result for the largest mesh by the mesh-based method and (f) elimination result for the largest mesh by the stroke edge-based method.

The 376 small meshes consist of 365 stroke edge meshes and 11 stroke non-edge meshes. For the 365 stroke edge meshes, the number of meshes whose elimination result is the same under the two different methods is 281, accounting for 77% of the total meshes, in which the eliminated segment of the mesh is just an edge segment, which is the reason for the same elimination results. The number of meshes whose elimination result is considerably different under the two different methods is 84, accounting for 23% of the total meshes, in which the eliminated segment of the mesh is not an edge segment, which is the reason for the different elimination results. The areas of these 54 stroke edge meshes lie within the range of [17489.9 m^2^, 74251.3 m^2^]. Hence, this paper selects the smallest (17489.9 m^2^), modest (31196.5 m^2^) and largest meshes (74251.3 m^2^) as typical regions to visually compare the elimination results of these two different methods, as shown in Fig 8.

Fig 8 reveals that the differences among the three typical regions for the stroke edge meshes are as follows:

For the three typical regions of the smallest, modest and largest meshes, the mesh-based method eliminated road segment b because it is the least important road segment. As a result, the road stroke where road segment b was located was split in the middle, and the road was divided into two road strokes, as shown in Fig 8A, 8C and 8E. In contrast, the stroke edge-based method used edge segments as the elimination units, and it preserved road segment b and eliminated edge segment a. Therefore, the road strokes where road segments a and b were located were both preserved, the visual continuity was much better, and the overall structure of the road network obtained by the stroke edge-based method was more similar to that of the elimination result of the human cartographer, as shown in Fig 8B, 8D and 8F, respectively.

**Fig 9:**
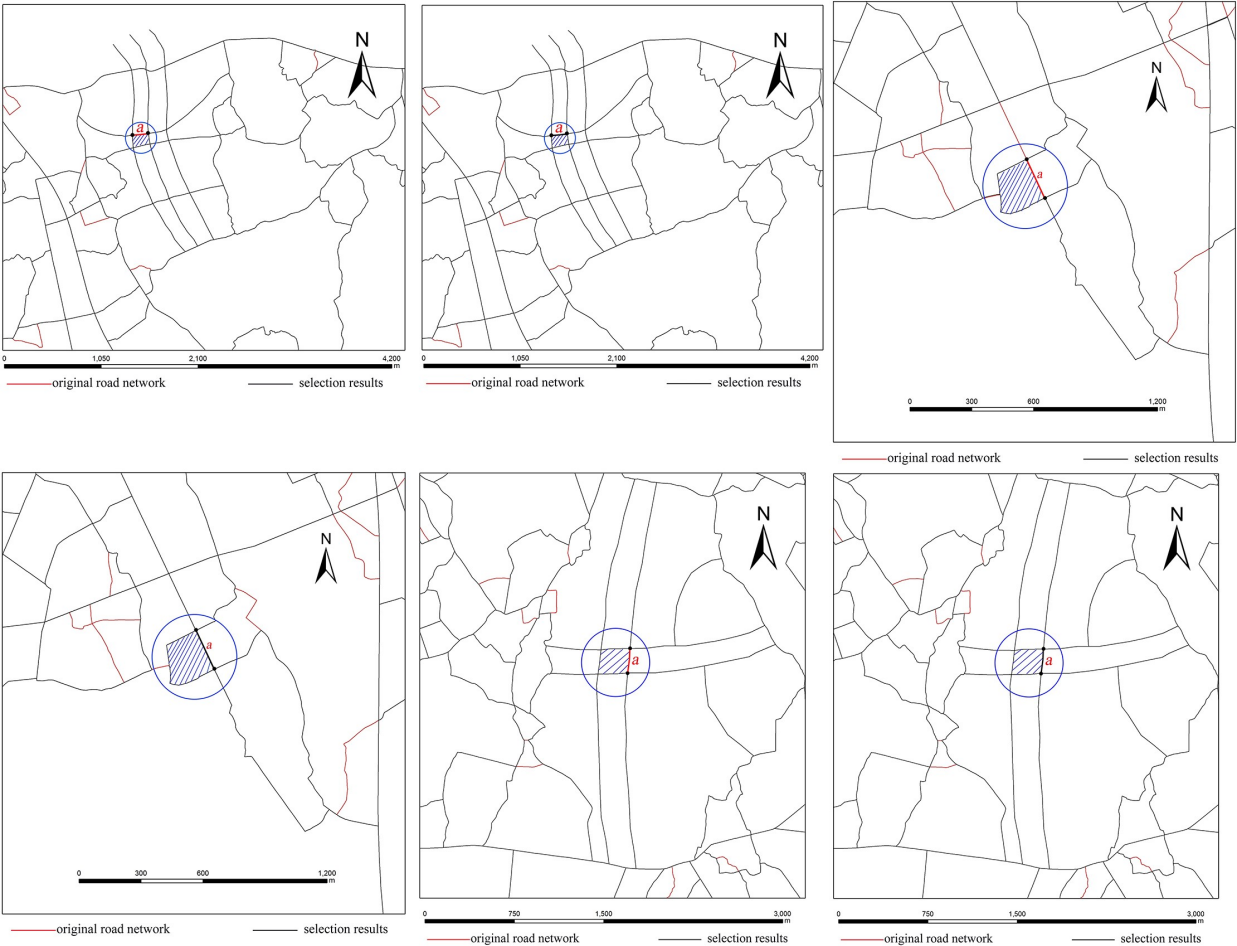
Comparison of the three typical areas for the stroke non-edge meshes with different elimination results. (a) Elimination result for the smallest mesh by the mesh-based method, (b) elimination result for the smallest mesh by the stroke edge-based method, (c) elimination result for the modest mesh by the mesh-based method, (d) elimination result for the modest mesh by the stroke edge-based method, (e) elimination result for the largest mesh by the mesh-based method and (f) elimination result for the largest mesh by the stroke edge-based method.

In addition, the elimination results by the two methods are quite different for each of the 11 stroke non-edge meshes, their areas lie within the range of [22689.6 m^2^, 49956.1 m^2^]. Hence, this paper selects the smallest (22689.6 m^2^), modest (39498.1m^2^) and largest meshes (49956.1 m^2^) as typical regions to visually compare the elimination results under the two different methods, as shown in Fig 9.

As shown in Fig 9, the differences among the three typical regions for the stroke non-edge meshes are as follows:

For the smallest, modest and largest three typical regions, it is clear that the stroke non-edge meshes play an important role in the preservation of the road stroke connectivity and spatial structure of the road network. The mesh-based method eliminated road segment a because it is the least important segment. As a result, the road stroke where road segment a was located was split in the middle, and the road stroke was divided into two road strokes, as shown in Fig 9A, 9C and 9E. In contrast, the stroke edge-based method did not eliminate any road segments in mesh I, so the road connectivity and spatial structure of the road network were both preserved, as shown in Fig 9B, 9D and 9F.

## Conclusions

The mesh-based method is a common, advanced and powerful mesh elimination method in which small meshes are sequentially processed and the road segment with the least importance in each mesh is eliminated. However, the connectivity and integrity of road strokes may be destroyed in specific regions by this method because certain road segments located in the middle of the road strokes could be eliminated. To address this issue, this paper proposed an elimination method for isolated meshes in a road network considering stroke edge feature, which can not only preserve the spatial structure and density characteristics of road networks but can also maintain the connectivity and integrity of the road strokes themselves. The following conclusions were obtained from the experimental validation and analysis using actual data:

1. For the eliminated meshes, all small meshes were eliminated by the mesh-based method. In contrast, only stroke edge meshes were eliminated by the proposed method, and the stroke non-edge meshes were preserved directly. Additionally, the elimination order determined by the proposed method also differed from that determined by the mesh-based method.
2. In terms of the quantitative analysis, by comparing three basic statistical indexes, the distribution density of the road meshes in the results obtained by the proposed method is closer to that of the road meshes in the 1:50,000 standard map. The modified average connectivity of the results by the proposed method is 1, which demonstrates that the connectivity and integrity of all road strokes are preserved. In addition, compared to the elimination result of the mesh-based method, the maximum similarity between the result of the proposed method and the standard map increases 2%.
3. Regarding the visual analysis, 77% of the elimination results for the stroke edge meshes are the same under these two different methods, where the eliminated segment of the mesh is just the edge segment. For the remaining 23% of the elimination results, where the eliminated segment of the mesh is not an edge segment, the elimination result for each mesh under these two methods is different. The stroke non-edge meshes of the small meshes are directly preserved and need not be eliminated, which is quite different from the mesh-based method.

Future research should focus on two aspects: first, the proposed method addressed the road elimination problem for small meshes, but it did not consider the spatial distribution characteristics of the aggregated small meshes, and therefore, future work should focus on the identification and elimination of the aggregation pattern of small meshes. Second, the proposed method needs to be applied to a wide range of scales to further examine its applicability.
